# Needs Detection for Carers of Family Members with Dementia

**DOI:** 10.3390/healthcare10010045

**Published:** 2021-12-27

**Authors:** Oriol Turró-Garriga, Maria del Mar Fernández-Adarve, Pilar Monreal-Bosch

**Affiliations:** 1 Dementia Registry of Girona (ReDeGi), Institut d’Assistència Sanitària, 17190 Salt, Catalonia, Spain; 2 Ageing Disability and Health Research Group, Girona Biomedical Research Institute, 17190 Salt, Catalonia, Spain; 3 Hospital Reina Sofia, 31500 Tudela, Navarre, Spain; mm.fernandez.adarve@navarra.es; 4 Psychology Department, Education and Psychology Faculty, University of Girona, 17004 Girona, Catalonia, Spain; pilar.monreal@udg.edu; 5 Ageing, Culture, and Health Research Group, University of Girona, 17004 Girona, Catalonia, Spain

**Keywords:** Alzheimer’s, dementia, carer, needs, resources, mental health

## Abstract

Aim: To determine the perceived needs of carers of non-institutionalized family members that suffer from dementia. Methods: Two-steps qualitative study by focus groups of relatives in three centres of different characteristics from the Girona Health Region (step 1) and two in-depth interviews with significant professionals in dementia care (step 2). The analysis was performed based on the interpretation of the transcribed data and the bottom-up coding of categories and themes. The information was triangulated and coding was agreed upon. Results: There were three groups, 26 main carers of community-dwelling relatives with dementia in step 1 and two in-depth interviews with dementia-specialised healthcare and social care professionals in step 2. The demands were categorised according to three main themes: whether they were addressed to the members of care services for more direct and close care, to the agencies for a better joint working and less fragmented system, or to society for better comprehension and social recognition. We emphasize the need for a consultation-liaison reference figure throughout the process both for aspects of greater efficiency in the management of resources and for greater empowerment of carers.

## 1. Introduction

**Figure 1:**
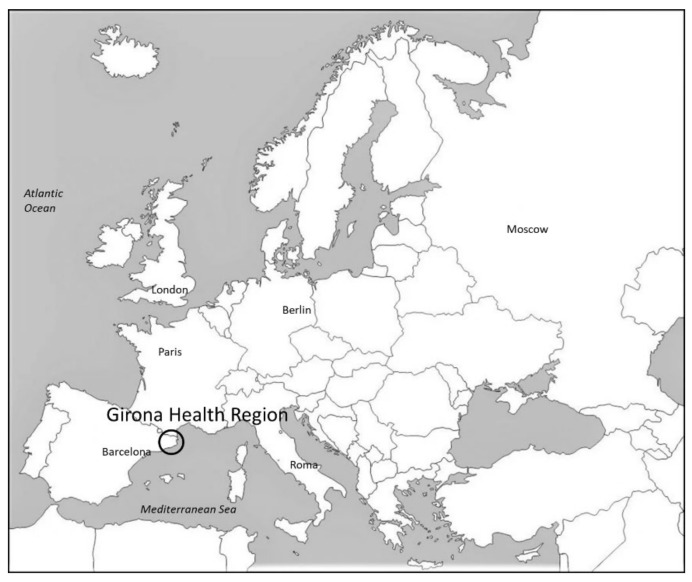
Localisation map of Girona Health Region.

Since 2007, the Registry of Dementia of Girona (ReDeGi) has registered the new cases of dementia diagnosed in the different hospitals of the Girona Health Region, a Mediterranean semi-urban region (137 h/km^2^) in the Northeastern part of Catalonia (Figure 1), reporting a clinical incidence of 6.6 cases per 1000 persons/year [1]. From the ReDeGi data, we know that at the time of diagnosis, people with dementia live mostly in their own home or in that of a relative [1,2]. Even so, differences are observed in the use of health resources when the context is more rural and older or younger and more urban [3].

The effects on physical and mental health of providing care to a family member with dementia have been widely described [4,5,6]. Numerous studies quantify, describe, and delineate the consequences of dementia care, the costs involved, or the effect of interventions [7,8,9]. Most of the studies quantify the burden report, the anxiety, or depression related to care, but few studies have asked them what could enhance their experience or what barriers have been found in the process of care. Some authors tried to bridge the gap linking this perception with the existence of unmet needs (due to existing resources). Therefore, to facilitate actions to be taken, needs have been classified as “in need of”: (a) information on the illness; (b) training for the management of activities of daily living and behavioural disorders; (c) emotional support; and (d) access to formal resources. Other authors have grouped them in “patient management needs” (information, behavioural disorders, formal and informal support, etc.) and “personal carer needs” (physical and emotional health, own-life management, etc.) [10,11]. A recent study has identified three key aspects for the adaptation of carers: (1) the stage of dementia, (2) the context of the carer, and (3) the context of where care takes place [12].

Despite this, the evidence of the self-experience of carers of a person with dementia research is still lacking, most of all in post-diagnostic care and services support. Fortunately, qualitative studies about carers’ perception are rising world-wide [13,14]. Recently, two reviews have been published, and one from the UK identified four main themes: information for carers, process of diagnosing dementia, difficulties accessing support, and cultural differences of experiences of services [15]. In a similar way, the second review from Spain tried to identify the barriers for taking part in interventions as carers. This study shows some interesting barriers of carers, such as difficulties adapting to the intervention’s schedule due to a lack of time or incompatibility with its dates or timetables, difficulties with accessibility, or impossibility to separate from family members to attend due to the absence of relief or a feeling of guilt [16].

Based on the different existing information about self-expressed needs of carers of individuals with dementia, a study was proposed with the aim of identifying the needs perceived by the caring relatives regarding the provision of care and the care received from the healthcare services. More specifically, a distinction was made between needs arising from care, the use of healthcare and/or social resources, and personal needs.

## 2. Material and Methods

### 2.1. Design

Exploratory and descriptive study based on qualitative methodology.

### 2.2. Participants

The participants were primary carers of a non-institutionalized relative diagnosed with dementia, recruited within the framework of ReDeGi. The study’s researchers highlighted the need to incorporate the participation of different carer profiles based on gender, kinship, or urban/rural habitat. The heads of each centre incorporated relatives of people with dementia in different stages of the disease, taking into account the previously decided sample criteria and thus maintaining the anonymity of the person with dementia for the researchers of the study. Relatives were recruited in a nursing home daycare centre (GF_B), in a geriatric and post-acute care centre (GF_F), and in a neurology service (GF_S). All participants speak and know Catalan and Spanish, and everyone spoke whichever made them feel more comfortable.

People with limited mobility or hearing impairments were excluded from the selection.

### 2.3. Procedure

The study consisted of two different steps. The first one was centred in giving a voice to the carers and collecting relevant information about their perception of healthcare and social care support systems. The second one was to face carers perceptions with high-expertise professionals from healthcare and social care systems.

Step one consisted of three focus groups of convenience sampling, and the second step was formed by two in-depth interviews with reference professionals to complement the information we already had from the focus groups [17]. The focus groups were conducted by O.T., helped by one representative of each centre for gathering additional information. Groups were formed from June to October of 2018 and then analysed and reported in 2019 [18]. The second step had been scheduled by early 2020, but the COVID-19 pandemic delayed it until the spring 2021.

The guide of the main topics to be addressed to focus groups was developed from the analysis of the bibliography and clinical experience (Table 1) [19,20,21,22,23]. The focus groups were recorded (audio) and transcribed for analysis. The study was approved by the Research Ethics Committee—CEI reference Girona cod. 2017.138 protocol v2: 23/10/2017. Before forming the groups, all participants signed the informed consent to record the session and process the data. O.T. did the transcriptions, and all personal information (names, addresses, etc.) was not included, keeping the confidentiality of the data. At a later stage, the in-depth interviews were conducted with reference professionals in the field of health (a neurologist specializing in dementia) and social welfare (social services psychologist in care for the elderly) to complement the information.

After the content analysis of focus groups (step one), in-depth interviews were conducted (step two). The aim of these interviews was to focus on the view of the professional members of family-carers who reported needs in their caring role and their experiences with health and social integrated care support services. Furthermore, these interviews helped to build up more information and knowledge about the dementia care process.

### 2.4. Analysis of the Data

The analysis is based on textual data to arrive at the theory [24]. The analytical procedure was divided into two distinct and not necessarily sequenced stages: the textual level and the conceptual level. The transcription coding strategy was bottom-up in the first stage. In the second, the conceptual level, the analysis was organized by relating and systematizing the quotations and codes in categories.

Finally, triangulation of researchers was used as an analysis strategy and control mechanism in the rigour of the research. The authors performed the data analysis independently and then triangulated the information. The computer program Atlas-ti version 8.1 was used for the analysis.

## 3. Results

Three focus groups were formed from a total of 26 people and two in-depth interviews with significant professionals. Table 2 presents the main characteristics of the sample by focus group. Three sessions were conducted for the triangulation analysis, reaching a high degree of agreement on the number of quotations and the adequacy of the categories and topics.

### 3.1. Classification of Carers’ Needs

The obtained information from focus groups was classified in 158 quotations, 60 codes, and 27 categories. Categories were stratified into three different themes based on a hierarchical approach of duties on integration services: (1) members of the services, (2) services and organisations, and (3) agencies and policies. Table 3 shows the classification of categories into each theme.

Moreover, this information was faced with two highly experienced professionals who linked it with the demands they usually receive. In that case, these professionals highlighted demands for proximity, demands of easy access to the administration, and a social demand of some recognition by society for caring for its older people with dementia.

#### 3.1.1. Demands for Services; Demands for Proximity

General practitioners (GP) and primary social care services received the highest number of demands related to unmet needs. These unmet needs were related to the diagnostic process and clinical follow-up, coordination between professionals, adequacy of information, and proximity. This proximity or engagement was reflected in carers who referred to a wide-ranging relationship with primary care professionals as opposed to those with whom this relationship did not exist or was more distant. In the first case, professionals perform the functions of a catalyst for demands, unlike when assistance is split between different professionals and services (GP, specialised care, social services, insurance healthcare, etc.).

Presence of a single referent: *GF_B_P3: In the case of my mother, in the early stages, she was very well cared for in the village; moreover, as many years ago, the same doctor was the one who realized before the family that something was wrong. She was then diagnosed and treated at the hospital by a neurologist, but the explanations and answers were almost always resolved by the village doctor. Over the years, there has been a more trusting relationship than with the neurologist, who we only saw once a year!*

Absence of a single referent: *GF_S_P6: In my case, the following-up stopped. I was told that the neurologist had retired and that I had to do the following-up with the primary care physician and that if we detected something, we should ask for another appointment. When this happened, I was told that the file had been closed and that I had to go on a waiting list of at least three months. I see this as absurd, how can a case be closed when the person has not stopped having the illness and will need more and more help?*

#### 3.1.2. Demands for Better Organisation

The demands to the services and organisations were focused on the accessibility of resources or the lack thereof, the fragmentation of the services, the territorial equity of available resources, the lack of specific training for carers. and the need for personalized emotional support to care for themselves.

The professionals interviewed were the first to highlight the lack of joint working, the limitations in the relationship between health and social services when it comes to collecting and sharing information, coordinating actions, adapting resources to needs, and adjusting them to the abilities of the person and their environment. For their part, the carers emphasized the fact that they had to repeat the same information about their needs when contacting social services or health services given the lack of integration of information systems between these services despite all of them being publicly owned services. These are difficulties that in turn incurred the desire to assume greater responsibility and participation in the process as another meaningful asset.

Professional health services: *Lack of complementarity, this would be an aspect to work on, and very seriously, both with primary care and with social services. So that people receive the best care close to their homes and do not always have to go to the hospital. There is administrative coordination, but I have been here for more than 25 years, and the changes are very small.*

Professional social services: *I think there should be a single reference for each family. In theory, we already have a circuit, a continuum, but it is not being done, and this tires families, demoralizes them, and sometimes they end up not arriving. The “call here, call there” is still very common.*

#### 3.1.3. Demands beyond Care

Finally, a set of demands with a more social dimension emerged. Demands related to the agencies and polices for a greater social understanding of the role of carers and the recognition of the social task they perform. There were also demands related to a more positive image and better care for the older adult in dementia care centres and in society. Demands of facing the stigma that remains both in society and in services and professionals that might take care of them. Occasionally, reference was also made to the legal and bureaucratic difficulties associated with a changing process that sometimes calls for greater agility in procedures and ease in decision-making.

Social recognition of the carer: *GF_F_P2: It’s that sometimes not even your own family understands you or understands what you do. You explain it, and they don’t understand it, not my brother, my uncle … and they also tell you ‘but if I see him very well, it’s very good’ and you think…*

Assignment of resources: *GF_S_P7: First thank you, I have to congratulate you for the work you do, because holding Kleenex tissues is not easy, but you have to find more ways (…) How am I going to ask for a psychiatrist for my mother if we don’t even have diapers! How expensive they are! And I have to go and ask for them from the Red Cross, here, there. All day crying out for diapers … and as I have to change my mother constantly. And it is a social issue, not just medical.*

**Figure 2:**
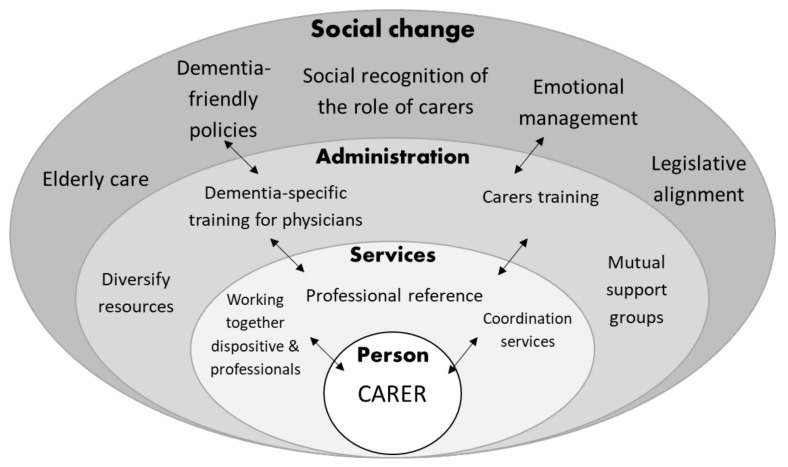
Representation model of the categorised demands and expressed needs.

For a better illustration of unmet needs expressed by carers, we developed a model. Figure 2 shows how carers explain the relationship among their own needs on a multi- and interrelated level. The authors decided to situate the person, the carer, at the bottom of the figure even though it could be read as the core of the debate, as suggested by interviewees.

### 3.2. The Reference Professional: A Key Transversal Solution

After this two-step analysis, according to which level they were addressed, the perception of specialists configured one transversal approach. Carers and professionals showed high concordance in the analysis of the situation and the proposed solutions. The highest exponent of these was the claim for a consultation-liaison professional, a key reference: a person, a figure who may have different professional profiles, but who, in any case, must be accessible through a range of channels and who accompanies the carers through the different moments and stages of the illness.

**Figure 3:**
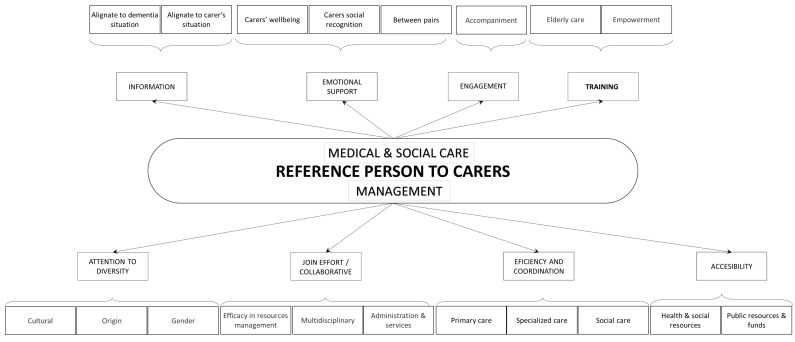
Figure of references’ attributions.

Having recollected the different needs related to this professional figure, we see that there is a group of demands regarding management support and another regarding care needs. Table 4 shows the themes, categories, and quotations related to the demands. In addition, in Figure 3, we draw the needs expressed concerning this liaison figure, an element included in care system who has a personal relationship with the carer; who acts as a link between the person and the different services, professionals, and resources; who is more accessible than other professionals and accompanies them in the process of the illness and in the decision-making process.

## 4. Discussion

The aim of this study was to determine the needs perceived by family carers of people with dementia cared for at home in the Health Region of Girona (Figure 1), That is, to give a voice to those people who, without having a definite “diagnosis as a carer” or having their own business concerning care, are the ones who bear the greatest care responsibilities.

Carers have expressed their needs, some related to members of the healthcare system, others with access to resources, etc., and all of them may be fully or partially not covered. This gradation also correlates with the perception of one’s own well-being or discomfort and, consequently, with the type of care them can be given [25]. From the set of results obtained, we observed how different levels of needs were mainly identified depending on who the requests were addressed to (services, administration, and society). At the same time, it was observed that two different types of demands were channelled through one figure: those related to management and those related to the person’s care.

### 4.1. The Adequacy of Services

The first group of needs was that of an adaptation of the health and social care to be more transversal, including efficient joint-working and continuity throughout the process. Demands comprised the inclusion of the person’s environment as an essential part of care, and emphasized the support, follow-up, and coordination between the different care services. Widely expressed needs include aspects of planning, emotional management of patients and carers, social intervention, etc.

Demands associated with situations of change or crisis are those situations when finding effective channels that provide answers is more necessary, a situation that in daily practice remains unresolved and that was closely linked to the reference figure. Moreover, as one participant pointed out, this figure could be important because she/he is up to date on how the person is. This is a demand that is widely observed in different European countries [26].

The figure of reference or case management requires greater flexibility of services and interrelationship between agencies as well as the training of the person in the different areas of health and social care [27,28], which is demand associated, on the other hand, as a reflection on the lack of flexibility of the entities and assistance services, which are more centred on the time management of their own operation than in the needs of the person [29]. In this sense, previous studies have observed that in rural areas. there is a greater perception of lack of support [30] although not all studies are conclusive and identify other factors as the major differences [31].

### 4.2. The Empowerment of Carers and Their Environment

The second group of needs are related to accompanying carers in the care process as an active part in the process. A total of 85.7% of people with dementia are cared for at home or by a relative [1]. A study by IMSERSO (The Institute for the Elderly and Social Services) stated that in our context, 50% of carers consider that they have no choice but to take care of the person, and 46% indicate that, due to their economic situation, no alternative can be considered [32]. In this sense, the needs were for information and training as well as health literacy, and these are appropriate to the situation and the context of specific care of the person [33,34]. Lack of sufficient tools and support can lead to burden report in carers. The burden, which carries the risk of giving up, depends on many factors, such as the type and severity of symptoms, the duration of dementia, personal characteristics of the carer, and the support he/she receives from health resources and the family environment [35]. In all these situations of stress and burden, the morbidity and mortality of carers increase significantly [36]. Problem-focused coping strategies, self-esteem, or resilience are proven strategies for the better mental health of carers [37].

The objectives of the study also contemplated identifying differences according to the contextual characteristics of the care. Thus, differences or discrepancies were observed between the development of the care task in the rural or urban context, between men and women, and between couples and children. In more rural or isolated settings, participants referred to greater difficulties in accessing both health and social resources. However, beyond the rural or urban context, the existence of different cultures within the region and with different ways of life, values, and different ways of doing things also appeared in the interviews with professionals.

The main unmet needs and demands were similar by both men and women. However, women mostly undertake the care of relatives with dementia. Some of the male participants confirmed some help by other female family members; meanwhile, husbands presented differences in being the ones who mostly assume the roles of sole carers. Younger people, despite not ceasing to be referents, prioritize or use more external resources or services. This reality, previously described in the literature, is also beginning to be seen among young daughters due to the need to maintain employment [38].

Figure 2 shows a graphical representation of the model of demands recorded by carers. These demands go beyond the care of dementia and their own needs, of health care, and social resources but also are a direct appeal to society to continue to participate in society, and this includes people with dementia, as already described above [39]. Changes in the professional assistance and vision of the illness reflect a more positive and constructive attention. In this sense, some of the participants described the need to combat the social stigma that dementia still entails today. Therefore, some participants made a demand for support groups and/or community spaces where, beyond the specific training on the management of the illness, interaction is possible between the carers or with health professionals. A need that also arose was the feeling of isolation and social misunderstanding that caring for a family member with dementia generates. A collective demand for a change of view of people with dementia and their personal environment, to generate social support and translate into a reduction in the burden and an increase in the quality of life and mental health of both those with dementia as well as their family carers [5,40].

### 4.3. Limitations

The literature contemplates different ways of approaching the management of the needs of carers although there is no single measure to do so, and there is no consensual instrument to determine these needs or which areas it should include [23,41]. One objective of this work was to include the vision of families living in both rural and urban environments although the city of Girona, with one hundred thousand inhabitants, is the largest in the province. Therefore, the use of the Girona Dementia Registry as the reference area may limit its external validity in some respects, especially in relation to the metropolitan areas of large cities; however, the range of services is (or should be) comparable, at least, to the whole of Catalonia and similar to the surrounding regions. In addition, the set of information and analysis derived from the register itself allows us to complement the description and composition of the study area. Another limitation of this study is given by the focus group method itself and the set of profiles. This study was split by the beginning of the COVID-19 pandemic in early 2020. Although we asked about this point in in-depth interviews, no differences in structural needs were reported. Moreover, some carers applaud the virtual and telephonic follow-up visits and did not have to move the relative to the hospital. However, this study lacks the voice of other carers who, due to the very need to be providing care or the fact of having a more distant relationship with care services, are not represented in this work. Even so, the needs exposed cover three different contexts with people in different situations and heterogeneous realities that show and contrast a reality shared by the majority.

### 4.4. Key Points and Future Lines of Study

This study attempts to cover the lack of reference, especially in our context, to the opinion and perception of unmet needs of carers of non-institutionalized relatives with dementia. Summarising the carers’ demands, we could identify four main themes. Carers claim the need for more direct and close attention, for better joint working among services, a less fragmentated system, and they also ask to continue to belong to the community with a better comprehension and recognition by health and social care systems but also by society as a whole.

Giving people the opportunity to express themselves allows us to identify aspects that are otherwise difficult to record. Attitudes, demands, and actions that could be difficult to record in the medical records of patients can still have a great impact on the person even on the dementia process as well. Thus, cultural notes were pointed out that must be analysed and, as we could see in some of the carers, the use of a sense of humour as an interesting coping strategy or communication tool [42]. Therefore, more studies are needed that give voice to the person at the centre, and if, in this study, the voice of the carer has been prioritized, there are always silenced voices, and it will be our duty to leave no one behind.

## Figures and Tables

**Table 1:** Focus group script.

1. The main deficiencies in the care received by the medical and assistance teams.
2. Difficulties observed in information management.
3. Use of care resources for supporting carers, the family accompaniment, and support groups.
4. People, entities, and/or groups that have given them the most support when facing care.
5. Main difficulties in carrying out daily life.

**Table 2:** Descriptive analysis of the sociodemographic of the participants by discussion group.

	DG_B (n = 9)	DG_F (n = 8)	DG_S (n = 9)
**Sex—female**, n (%)	9 (100)	6 (75.0)	2 (22.2)
**Age**, mean (DE)	63.4 (8.8)	54.4 (7.2)	65.8 (11.8)
**Kinship**, n (%)			
***Spouse***	1 (11.1)	0 (0.0)	6 (66.6)
***Adult-child***	6 (66.6)	6 (75.0)	2 (22.2)
***Other relative***	2 (22.2)	2 (25.0)	1 (11.1)
**Scholarship**, n (%)			
***Low***	0	0	1 (11.1)
***Primary***	2 (22.2)	3 (37.5)	3 (33.3)
***Secondary***	5 (55.6)	3 (37.5)	2 (22.2)
***Degree or higher***	2 (22.2)	2 (25.0)	3 (33.3)
**Cohabitation—yes**, n (%)	3 (33.3)	5 (62.5)	8 (88.9)
**Context—rural**, n (%)	8 (88.8)	2 (25.0)	3 (33.3)
**Active job**, n (%)			
***No***	5 (55.6)	4 (50.0)	8 (88.9)
***Partially***	0	2 (25.0)	0
***Yes***	4 (44.4)	2 (25.0)	1 (11.1)
**Resources y services**, n (%)			
***LAPAD***	7 (77.8)	3 (37.5)	1 (11.1)
***Daycare centre***	4 (44.4)	3 (37.5)	3 (33.3)
***Domiciliary services***	3 (33.3)	4 (50.0)	1 (11.1)

DG_B, discussion group of Besalú; DG_F, discussion group of Figueres; DG_S, discussion group of Salt; LAPAD, public policy by dependence.

**Table 3:** Classification of categories into three main themes.

Member/Professional	Services and Organisations	Agencies and Policies
Accompaniment	Accessibility	Bureaucratic procedure
Clear and honest information	Professional of reference	Flexibility scheduling visits
Carer’s wellbeing	Involving carers in decision-making process	Social emergency resources
Emotional management	Education and training	Nursing home and residential resources
Guilty and negative perceptions	Psychological assessment	Equity between rural and urban areas
Economic burden	Working groups of mates	Facilitators to maintain caring at home
Carer’s social recognition	Adjusting the information to carer	Fragmentated care
Attention to diversity	Healthcare and social care systems coordination	Limited public resources and funds
	Primary and specialized healthcare coordination	
	Diagnostic process and post-diagnostic process discrepancies	
	Empowerment	

**Table 4:** Themes, categories, and quotations related with the figure of reference.

Theme	Categories	Quotations
**Attention**	Information	GF_F_P7: "*There is a need for a figure who you can have relatively direct access to and who can advise you both emotionally and with information, emergency management, logistics… I do not know if this is possible or if public health services could afford it…*”
	Emotional support	
	Creating a link/engagement	Healthcare professional: “*The carer must understand the illness very well to be able to manage everything that will happen to the person in the different stages. Therefore, the carer is a key element; if there is no carer, care is impossible, we have no other option*.”
	Training	
**Management**	Management of diversity	GF_B_P9: “*My husband takes care of her, gives her water, feeds her; in that case it helps me and my brother-in-law does the same… but, of course, if he has to change her diaper, they refuse, if she has to go to the bathroom, they won’t do it anymore… and I can’t leave the house for more than a few moments a day*.”
	*Cultural*	
	*Gender*	
	*Regional*	
	Joint working	Healthcare professional: “*Here, we need more complementarity, and this is an element that needs to be worked on, and very deeply, both with primary care and with social services. This is necessary to ensure that people receive the best care close to home and do not always have to go to the hospital. Because a bureaucratic coordination does exist, but the reality is that after 25 years working here, the changes regarding the attention to people are very small*.”
	*Between professionals*	
	*Between services and agencies*	
	*Between institutions*	
	Effective coordination	Social Services Professional: “*The referent should be a person close to the families who coordinates with the rest of the resources. I think there should be a single reference for each family. Ideally, there should be a single reference, both for social and health services. I would also say more proximity. Unfortunately, there is still a lot of ‘call this number, call that number’… Families find a lot of fragmentation among services*.”
	*Primary care*	
	*Specialized care*	
	*Social care*	
	Accessibility	*GF_S_P7: “First of all, thank you, I must congratulate you for the work you do, because holding tissues all day is not easy, but many resources are lacking (…) How can I ask for a psychiatrist for my mother if we don’t even have diapers! They are so expensive! And I have to go asking for diapers everywhere, to the Red Cross, here, there, … crying all day to get diapers… and I’m constantly changing my mother! In fact, this is a social issue, not just a medical one.”*
	*Resources*	
	*Funds*	

## Data Availability

The data presented in this study are available on request from the corresponding author.
